# The potential role of transcranial direct current stimulation in experimental ischemic stroke in adult male albino rats

**DOI:** 10.1038/s41598-026-51013-z

**Published:** 2026-05-18

**Authors:** Omar Ahmed Abdelbary, Nevert Farid Abdelsalam, Dalia Alaa El-Din Aly El-Waseef, Nevine El Nahas, Shaimaa Khedr, Walter C. Low, Andrew W. Grande

**Affiliations:** 1 Department of Histology, Faculty of Medicine, Ain Shams University, Cairo, Egypt; 2 Department of Neuropsychiatry, Faculty of Medicine, Ain Shams University, Cairo, Egypt; 3 Department of Neurosurgery, University of Minnesota, Minneapolis, MN USA

**Keywords:** Cathodal tDCS, Anodal tDCS, Ischemic stroke, Neuroprotection, Astrocytes, Microglia, Inflammation, Neurology, Neuroscience

## Abstract

Transcranial direct current stimulation (tDCS) is a noninvasive neuromodulatory technique with potential therapeutic applications in stroke, but the mechanisms underlying its neuroprotective effects in acute ischemia remain unclear. To evaluate the effects of cathodal versus anodal tDCS on neurobehavioral outcomes, histopathological changes, and inflammatory and glial responses in a rat model of focal cerebral ischemia. Adult male albino rats were randomized into normal non-ischemic, untreated stroke, sham, anodal, and cathodal tDCS groups. Neurological status and sensorimotor function were assessed 24 h after ischemia. Infarct volume (TTC), neuronal integrity (H&E and Nissl), and expressions of TNF-α, c-Fos, CD206, and GFAP were analyzed to characterize neuroinflammation, neuronal activity, and glial responses. Cathodal tDCS improved neurological scores and preserved sensorimotor function compared with anodal, sham, and untreated groups, the latter of which frequently exhibited acute coma. Histopathology in the cathodal group showed reduced necrosis, diminished inflammatory infiltration. In contrast, anodal stimulation produced only partial improvement, remaining significantly less effective than cathodal stimulation. Molecular profiling revealed that cathodal tDCS decreased TNF-α, enhanced astrocytic activation (GFAP) and neuronal activation (c-Fos), and promoted a trend toward M2 microglial polarization (CD206), whereas anodal tDCS exerted weaker effects. Cathodal tDCS conferred superior early neuroprotection and functional recovery after experimental ischemic stroke compared with anodal, sham, and untreated groups, likely through stronger modulation of inflammatory and glial pathways. While anodal stimulation showed limited benefit, cathodal stimulation demonstrated greater translational potential as an acute-phase intervention for ischemic stroke.

## Introduction

Stroke remains the second leading cause of death and a major contributor to long-term disability worldwide, representing a substantial global health burden. Ischemic stroke accounts for approximately 62.4% of all stroke cases, with millions of individuals affected annually and a significant proportion experiencing persistent neurological deficits and reduced quality of life. Despite advances in acute stroke management, access to effective therapies remains limited in many regions, particularly in low- and middle-income countries^1^.

In the Middle East, Egypt reports one of the highest incidences of stroke, reflecting a considerable public health challenge driven by population aging and the increasing prevalence of vascular risk factors. These factors underscore the urgent need for accessible, safe, and cost-effective therapeutic strategies^2^.

Clinical studies have shown that a large infarct size can lead to mortality or significant morbidity within the first month post-stroke. Once irreversible damage occurs, large infarcts make recovery challenging. Consequently, reducing infarct size is critical in the treatment of acute ischemic stroke^3^.

No therapy reliably promotes post-stroke neuroregeneration. Despite robust preclinical data, translation of ischemic stroke therapies remains poor. Experimental approaches, including stem cell therapies and neuromodulation, showed preclinical promise but failed in large-scale trials^4^. Tissue plasminogen activator (tPA) is the only FDA-approved pharmacological treatment, limited by a narrow therapeutic window, short half-life, and risks of cerebral edema, hemorrhage, and blood–brain barrier disruption^5^. Endovascular thrombectomy and alternatives such as Tenecteplase offer improvements, yet recanalization and safety limitations persist^6^.

Globally, the utilization of intravenous thrombolysis (IVT) in acute ischemic stroke (AIS) remains markedly limited, despite ongoing advancements in thrombolytic therapies. Recent analyses highlight that access constraints, delayed presentation, and stringent patient-selection criteria continue to prevent a substantial proportion of eligible patients from receiving IVT^7^.

Nearly 94.2% of eligible AIS patients do not receive IVT, despite the availability of IVT and a rise in stroke units in Egypt. Many patients who are present inside the therapeutic window do not receive IVT because of in-hospital delays, and patients in rural areas are more likely to arrive at the hospital too late for therapy^8^.

Young adult acute ischemic stroke patients in Egypt often experience high rates of post-stroke depression and face challenges in their ability to work and support their families^9^.

As a result, a significant proportion of patients survive with severe neurological deficits. Rehabilitation for these patients typically focuses on physical, occupational, and speech-language therapy, as well as preventing medical complications^4^.

Transcranial direct current stimulation (tDCS) is one of the non-invasive brain stimulation techniques that have transformed neuropsychiatry by offering safe and well-tolerated alternatives to invasive procedures^10^. The potential effectiveness of tDCS in modifying plasticity in both patients and healthy individuals has attracted growing attention. To take advantage of the cortical excitability-modifying effects, a weak direct electric current is passed via the scalp. By altering membrane polarity, it mainly modifies cortical excitability. While cathodal stimulation is thought to result in hyperpolarization and decrease cortical excitability, anodal stimulation is thought to promote neuronal membrane depolarization and increase cortical excitability^3^.

There is accumulating evidence supporting the clinical efficacy of tDCS in treating conditions such as Alzheimer’s disease, Parkinson’s disease, chronic depression, and compulsions related to food, drugs, and alcohol^11^. In stroke rehabilitation, tDCS has shown promise in improving functional outcomes by inducing long-term neuroplasticity changes and modulating local and distant networks that underlie various clinical symptoms resulting from stroke^9^.

Compared to other brain stimulation modalities such as transcranial magnetic stimulation (TMS) and deep brain stimulation (DBS), tDCS is relatively simple, low-cost, and associated with fewer risks, making it more feasible for widespread clinical and outpatient use, particularly in resource-limited settings^12^.

tDCS could be a promising non-invasive tool in the early stages of brain ischemia. However, despite its widespread use in clinical settings, little is known about the molecular mechanisms and microscopic changes underlying the beneficial effects of tDCS^13^.

Previous studies have demonstrated the neuroprotective potential of transcranial direct current stimulation in experimental stroke models, including the effects of cathodal stimulation. However, many of these studies have focused on single-polarity stimulation approaches, with stimulation applied at delayed time points and often involving repeated sessions^14,15^.

In addition, the literature remains inconsistent regarding the optimal polarity and timing of stimulation, with variable outcomes reported across different experimental conditions. Importantly, studies evaluating the effects of tDCS in the hyperacute phase of stroke are limited, and direct comparisons between cathodal and anodal stimulation under identical conditions are scarce^14,15^.

This study aims to evaluate the effects of cathodal versus anodal tDCS on neurobehavioral outcomes, histopathological changes, and inflammatory and glial responses in a rat model of focal cerebral ischemia. By investigating these aspects, the research seeks to uncover the mechanisms behind the clinical benefits of tDCS observed in human studies and to inform the development of optimized tDCS protocols for use in human patients.

## Materials and methods

### Animals

Twenty-five adult male albino Wistar rats aged 10–12 weeks and weighing 200–230 g were obtained from and housed in the MASRI (Faculty of Medicine, Ain Shams Research Institute) Animal House, Faculty of Medicine, Ain Shams University (Cairo, Egypt). Animals were kept in clean plastic cages with stainless-steel wire lids under controlled temperature, humidity, and a 12:12 h light–dark cycle, with free access to chow and tap water. After a 7-day acclimatization period, all experimental procedures were conducted in the MASRI and the Histology Department Research Laboratory, Faculty of Medicine, Ain Shams University.

All experimental procedures were performed in accordance with the institutional and national ethical standards for the care and use of laboratory animals, and in compliance with the ARRIVE guidelines 2.0. Ethical approval was obtained from the Faculty of Medicine Research Ethics Committee, Ain Shams University (Ref. No.: FMASU MD287/2023; 12 October 2023).

### Experimental design

Animals were randomly assigned into five groups (*n* = 5 each):

1. **Normal non-ischemic**– no intervention.
2. **Untreated stroke** – right unilateral common carotid artery occlusion (RUCCAO) without further treatment.
3. **Sham tDCS** – RUCCAO followed by sham stimulation.
4. **Anodal tDCS** – RUCCAO followed by anodal stimulation.
5. **Cathodal tDCS** – RUCCAO followed by cathodal stimulation.

A total of five animals per group were included in the final analysis. Due to the high mortality rate associated with the acute ischemic stroke model, replacement animals were used when necessary to maintain consistent group sizes across all experimental conditions.

### Behavioral assessment

**Fig. 1:**
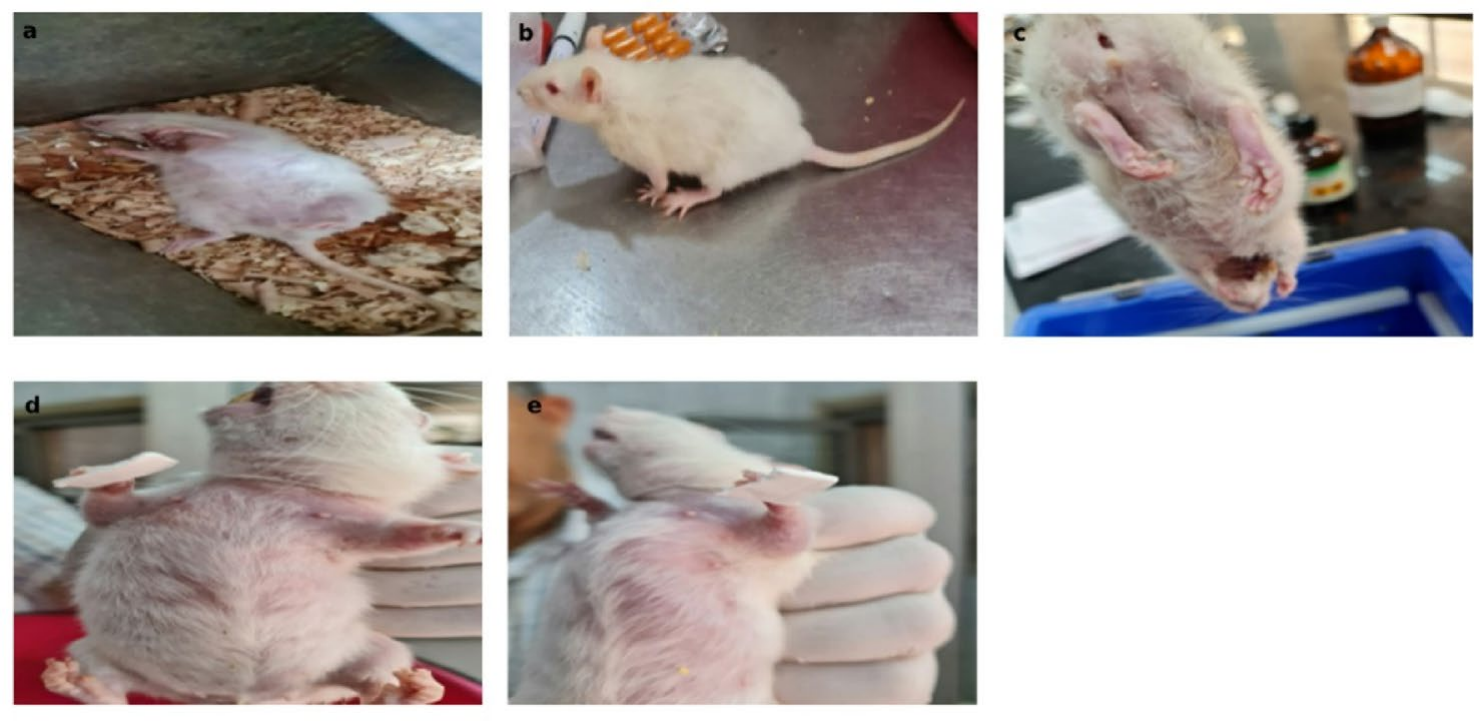
Rat exhibiting the absence of the righting reflex **(a)**. Rat demonstrating the presence of the righting reflex **(b)**. Rat showing a degree V ipsilateral paralytic deformity **(c)**. Rats undergoing the adhesive removal test **(d**,** e)**.

Behavioral evaluation was performed pre-surgery and 24 h post-ischemia as illustrated in Fig. 1.

A. All rats underwent behavioral monitoring to assess pain responses and righting reflexes as outlined in this methodology^16^. Rats’ levels of consciousness were categorized into six degrees (I to VI) based on their sensory and motor functions. Degree, I signified normal cage activity, while degree II indicated reduced activity. Degree III involved diminished activity coupled with motor incoordination. In degree IV, the righting reflex was triggered, causing the animals to stand up. In degree V, the righting reflex vanished, and animals reacted to pain stimuli. Finally, in degree VI, animals showed no response to pain stimuli. Rats that fell into degrees V and VI for a minimum duration of 30 min considered to be in a coma-like state.
B. The adhesive removal test, based on this protocol^17^, was conducted on all rats using double-sided tape. Both the contact time and removal time were recorded for each rat, with the test repeated three times and the best performance considered. The procedure began with a 30-minute acclimation period. After gently removing the rat from the testing box, a strip of adhesive tape was applied to the hairless part of the right forepaw. The rat was then returned to the testing box, and two timers were started, each running for a maximum of 120 s. The first timer recorded the contact time, defined as the moment the rat reacted to the adhesive tape by shaking its paws or bringing them to its mouth, indicating it had felt the tape. The second timer recorded the removal time, which ended when the rat used its mouth or the contralateral forelimb to remove the tape. The same steps were repeated for the left paw.

### Scoring system

To standardize results across animals, both contact and removal times were converted into a 3-point ordinal scale:

- **Score 1**: 0–60 s (baseline).
- **Score 2**: 61–120 s (moderate impairment).
- **Score 3**: 121 s (failed to complete the task).

### Induction of ischemic stroke

**Fig. 2:**
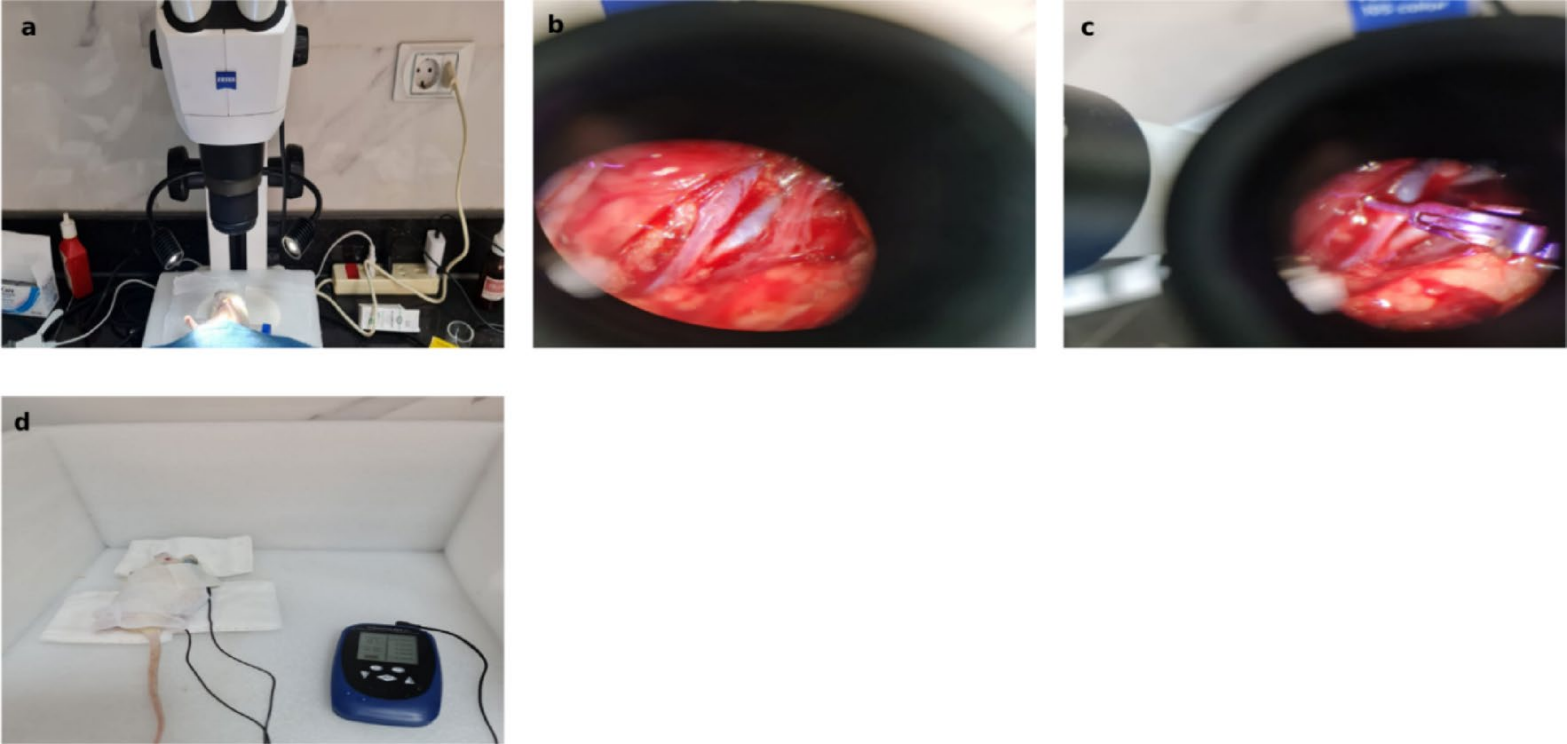
An anesthetized rat in a supine position under a surgical microscope **(a)**. The common carotid artery (CCA) exposed after blunt dissection, viewed under a surgical microscope **(b)**. The CCA occluded with a vascular clip for 90 min **(c)**. Rat receiving transcranial direct current stimulation (tDCS), with the active electrode placed on the scalp and the counter electrode attached to the trunk, secured with gauze **(d)**.

The RUCCAO surgery was conducted following the protocol established by Dr. Grande and Dr. Low at the Stem Cell Institute, University of Minnesota, and as described by^18^. This surgical procedure took place in the Histology Department Research Lab, Faculty of Medicine, Ain Shams University. The surgery involved several precise steps: all surgical tools were sterilized, and the rats were anesthetized with a ketamine and xylazine mixture. The rats were then positioned supine on a heating pad, and a midline incision was made on the neck to expose the right common carotid artery (CCA) through blunt dissection under a **dissecting microscope (Zeiss**,** Stemi 305 model).** The CCA was occluded with a vascular clip (**L-Aneurysmen Clip** ^®^, **Peter Lazic**) for 90 min, after which revascularization was achieved by removing the clip as illustrated in Fig. 2. Following the surgery, 5 mL of saline was administered intraperitoneally and subcutaneously to all operated animals. The neck incision was sutured, and post-surgical analgesia and antibiotics were given to ensure proper recovery.

### Transcranial direct current stimulation (tDCS)

Group IV rats received anodal stimulation, while Group V rats received cathodal stimulation. After being anesthetized, the rats were subjected to 20 min of tDCS (**TheBrainDriver® v2.1.)** on the cortical motor area of the ischemic hemisphere with a current amplitude of 0.5 mA, 20 min after surgery. Following the initial 20-minute stimulation, the rats underwent a 20-minute rest period, after which they received an additional 20 min of stimulation. The active electrode was placed on the rat’s scalp, while the counter electrode was attached to the trunk and surrounded by gauze as shown in Fig. 2 to prevent displacement. Group III rats received sham stimulation, which mimicked active stimulation but was turned off after 30 s^19^.

### Sample collection and tissue processing

Following RUCCAO surgery, the rats from each group were perfused at the twenty-four-hour mark. The perfusion procedure involved anesthetizing the rats and perfusing them with 200 ml of 5 mM sodium phosphate-buffered saline, followed by 500 ml of 10% buffered formalin^20^. After perfusion, the rats were euthanized via decapitation under ether inhalation anesthesia. Brain tissues were then harvested. The bodies of the euthanized animals were disposed of by incineration, in accordance with animal care guidelines and the ethical standards established by the Scientific Research Ethical Committee of the Faculty of Medicine, Ain Shams University.

### 2,3,5-triphenyltetrazolium chloride (TTC) staining

TTC staining was performed on one representative animal per group to visualize infarct localization. Due to the limited sample size, statistical comparison was not feasible, and TTC findings are presented for qualitative assessment only. Brains were rapidly cooled on ice, and four coronal sections were prepared and placed in glass Petri dishes containing TTC solution. Glass coverslips were applied to keep slices flat, and the dishes were incubated at 37 °C for 30 min. **Viable tissue stained red**,** while infarcted areas remained white**. Following incubation, TTC was replaced with 10% buffered formalin, and slices were fixed overnight. Stained sections were photographed within 3–7 days according to the protocol of^21^.

### Hematoxylin and Eosin (HE) staining and Nissl staining

Following this protocol^22^., H&E and Nissl staining were performed on four rats from each group. The brains were rapidly extracted, dehydrated in ethanol, and cleared in xylene. They were then embedded in paraffin, and 4 μm thick horizontal sections were prepared using a rotary microtome.

For H&E staining, the sections were immersed in hematoxylin for 3 min, rinsed under running tap water, and briefly destained in hydrochloric acid alcohol. After another rinse, the sections were dipped in eosin for 15 s, dehydrated through a graded series of alcohol, cleared in xylene, and mounted with coverslips. This method enabled the detection of histological changes. The stained slides were subsequently photographed.

For **Nissl staining**, sections were incubated in 1% toluidine blue at 45 °C for 30 min, differentiated in 75% alcohol, and rinsed briefly in distilled water. This technique highlights neuronal integrity by staining Nissl bodies **deep blue violet**, with the background tissue appearing pale, thereby allowing visualization of viable versus damaged neurons. Slides were subsequently photographed.

### Immunohistochemistry

Four rats from each group were subjected to marker analysis for tumor necrosis factor alpha (TNF-alpha) to evaluate the neuroinflammatory response. The brain tissues were processed and embedded in paraffin, then sectioned. The sections were deparaffinized in xylene, washed in 100% ethyl alcohol, and rehydrated in descending grades of alcohol. After washing twice in buffer, sections were incubated with 5% normal horse serum to block nonspecific staining, followed by another wash. Endogenous peroxidase activity was blocked using 3% hydrogen peroxide, and antigen retrieval was performed with citrate buffer. The primary antibody (Anti-TNF alpha antibody, ab220210, diluted 1:1000) was applied and incubated for one hour at room temperature. After washing, a secondary antibody (Goat Anti-Rabbit IgG, ab150077) was applied and incubated for ten minutes at room temperature. The sections were then counterstained with hematoxylin, dehydrated, cleared, and mounted. The stained slides were photographed. TNF-α–positive staining appeared **brown (DAB chromogen)**, while nuclei were counterstained **blue (hematoxylin)**, following established methodology^23^.

### Immunofluorescence study

Immunofluorescence was used to evaluate neuronal activation (c-Fos), astrocytic reactivity (GFAP), and microglial polarization (CD206) in brain sections from four rats per group. Brain tissues were formalin-fixed, paraffin-embedded, and sectioned coronally (5 μm). After standard dewaxing, antigen retrieval, and blocking, sections were incubated with primary antibodies (anti-c-Fos, anti-GFAP, anti-CD206) followed by fluorescently conjugated secondary antibodies and nuclear counterstaining with DAPI. Slides were mounted with antifade medium and imaged using a Leica DMI 6000 B fluorescence microscope. Each marker was acquired in a separate fluorescence channel, with **brighter areas reflecting higher marker expression**. Marker expression was quantified as the percentage of positive cells relative to total nuclei, enabling comparison across experimental groups^24^.

### Morphometric study

Morphometric analysis was performed to evaluate neuronal integrity, glial activation, inflammatory response, and neuronal activity across all experimental groups.

Quantitative analysis was conducted on the right cortical gray matter, excluding white matter, which was defined as the region of interest (ROI). For each animal, three frontal coronal sections were selected based on optimal tissue preservation and staining quality, and the entire cortical gray matter within each section was analyzed.

Histological and immunohistochemical staining (Nissl and TNF-α) were analyzed using QuPath software (version 0.6.0). Automated cell detection algorithms were applied with optimized threshold settings to quantify neuronal density and TNF-α immunoreactivity, expressed as numeric counts per defined field.

Immunofluorescence markers, including c-Fos (neuronal activity), GFAP (astrocytic activation), and CD206 (M2 microglial activation), were quantified using automated cell classification in QuPath. Positive cells were expressed as a percentage of total nuclei (DAPI-stained), and area percentage was calculated for comparative analysis.

For immunofluorescence analysis, image acquisition and quantification were performed in a blinded manner. Other histological analyses were conducted by a single investigator using standardized procedures.

The total analyzed area per animal corresponded to the full cortical gray matter across the selected sections. Image quality was routinely assessed to ensure adequate staining intensity, tissue preservation, and absence of artifacts that could compromise morphometric quantification. Only sections meeting these predefined quality criteria were included in the analysis.

## Results

### Behavioral observation

**Fig. 3:**
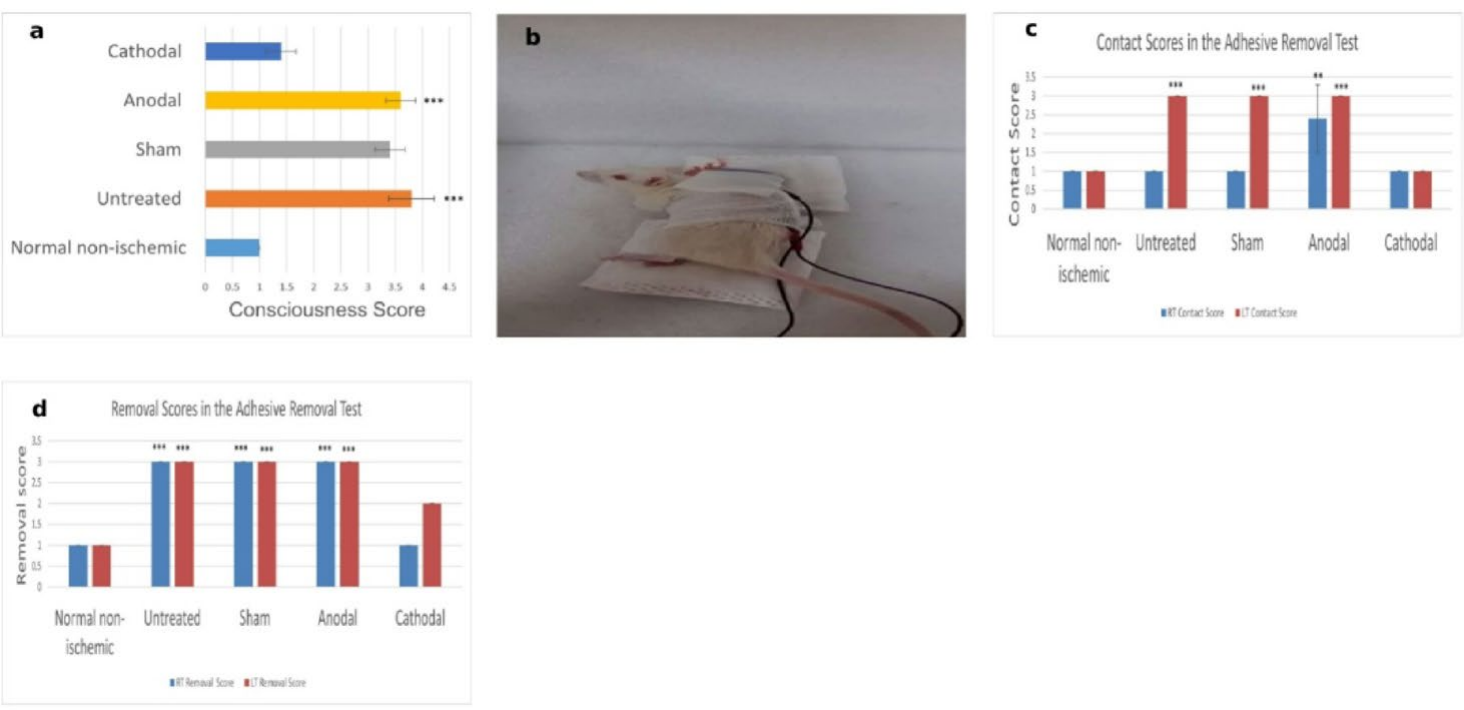
Functional outcomes across experimental groups following stroke and tDCS treatment: **(a)** Histogram of postoperative consciousness scores across groups (*** *p* = 0.0007264 vs. Normal non-ischemic). **(b)** Representative rat from the cathodal tDCS group regaining consciousness during the second stimulation session. **(c)** Histogram comparing mean adhesive contact scores after surgery, illustrating sensory recovery (***p = 0.00007987 (LT): Untreated, Anodal, Sham vs. Normal/Cathodal, **p = 0.001137 (RT): Anodal vs. all other groups). **(d)** Histogram of mean adhesive removal scores after surgery, demonstrating motor function performance (*** *p* = 0.00007987 vs. Normal non-ischemic).

All animals showed normal consciousness before surgery (Degree I). As shown in Fig. 3, after surgery, cathodal tDCS was associated with improved recovery, as reflected by lower median scores (1.4 ± 0.55) compared with the Anodal, Sham, and Untreated groups. The normal non-ischemic group remained unchanged (1 ± 0). Behavioral observations revealed hyperactivity and aggressive responses in several cathodal-treated rats, which also required higher anesthetic doses at euthanasia. Kruskal–Wallis analysis indicated significant intergroup differences (H = 19.174, *p* = 0.0007264), with post hoc tests showing significant differences between the Normal non-ischemic and both the Anodal and Untreated groups.

### Adhesive removal test

All animals exhibited normal motor and sensory function before surgery (Score 1). Post-surgery, sensory–motor performance varied across experimental groups. The Normal non-ischemic and Cathodal groups largely maintained function, while the Anodal, Sham, and Untreated groups showed marked deficits.

#### Contact time

After surgery, contact times were markedly different among groups (Table 1). The Normal non-ischemic and Cathodal groups maintained shorter contact times both before and after surgery. In contrast, the Anodal, Sham, and Untreated groups exhibited significantly prolonged contact times, with rats that failed to complete the test assigned a cutoff of 121 s.

On the RT side, the Anodal group demonstrated partial dysfunction (103.6 ± 28.65 s, Score 2), differing significantly from all other groups (H = 18.182, *p* = 0.001137). Sham and Untreated groups failed the test (20.0 ± 2.10 s and 18.0 ± 1.85 s, score 1 for those that completed, 121 s cutoff for failures), while the Cathodal and Normal non-ischemic groups maintained near-normal scores (1 ± 0).

On the LT side, the Normal non-ischemic group preserved normal function (4.00 ± 0.44 s, score 1), and the Cathodal group showed mild impairment (20.74 ± 2.10 s, score 1). By contrast, the Anodal, Sham, and Untreated groups exhibited severe deficits (121 s cutoff, Score 3). Kruskal–Wallis analysis confirmed significant intergroup differences for LT contact scores (H = 24, *p* = 0.00007987), with post hoc comparisons showing that the Normal non-ischemic and Cathodal groups differed from Anodal, Sham, and Untreated groups but not from each other.

These findings indicate that contact time, reflecting the ability to perceive and respond to tactile stimuli, was largely preserved in the Cathodal group, closely resembling Normal non-ischemic animals, whereas Anodal, Sham, and Untreated groups exhibited profound post-stroke impairments.

#### Removal time

Removal time analyses similarly demonstrated distinct group effects (Table 2). After surgery, the Normal non-ischemic group maintained near-normal removal times on both RT and LT sides (24.98 ± 2.50 s, Score 1). The Cathodal group showed only mild prolongation, particularly on the LT side (94.23 ± 9.42 s, Score 2.5).

In contrast, the Anodal, Sham, and Untreated groups failed to remove the adhesive in a timely manner, with all rats reaching the cutoff of 121 s and scoring 3. Kruskal–Wallis analysis confirmed significant differences among groups for both RT and LT removal times (H = 24, *p* = 0.00007987). Post hoc comparisons indicated that the Normal non-ischemic and Cathodal groups differed significantly from the other groups but not from each other, highlighting relative preservation of sensory–motor function in these two groups.

These results demonstrate that removal time, reflecting sensorimotor coordination and response, was maintained in Cathodal-treated rats, whereas Anodal, Sham, and Untreated animals exhibited severe deficits following ischemic injury.

**Table 1:** Contact time across experimental groups: Values are presented as mean ± SD. - Indicates test failure; cutoff value assigned to rats that failed to complete the test.

Group	Before the Surgery				After the Surgery				RT Sig*	LT Sig*
	RT Contact Time (s)	RT Contact Score	LT Contact Time (s)	LT Contact Score	RT Contact Time (s)	RT Contact Score	LT Contact Time (s)	LT Contact Score		
Negative control	4.66 ± 0.48	1.0 ± 0.0	3.12 ± 0.35	1.0 ± 0.0	5.12 ± 0.52	1.0 ± 0.0	4.00 ± 0.44	1.0 ± 0.0	a	a
Untreated	3.40 ± 0.41	1.0 ± 0.0	3.40 ± 0.41	1.0 ± 0.0	18.00 ± 1.85	1.0 ± 0.0	121.00 ± 0.0-	3.0 ± 0.0	b	b
Sham	11.70 ± 1.20	1.0 ± 0.0	4.58 ± 0.46	1.0 ± 0.0	20.00 ± 2.10	1.0 ± 0.0	121.00 ± 0.0-	3.0 ± 0.0	b	b
Anodal	6.87 ± 0.68	1.0 ± 0.0	7.14 ± 0.71	1.0 ± 0.0	103.6 ± 28.65	2.0 ± 0.0	121.00 ± 0.0-	3.0 ± 0.0	c	b
Cathodal	8.00 ± 0.82	1.0 ± 0.0	9.84 ± 0.95	1.0 ± 0.0	15.99 ± 1.60	1.0 ± 0.0	20.74 ± 2.10	1.0 ± 0.0	a	a

Groups not sharing the same superscript letter differ significantly.

RT Contact Score: H = 18.1818, *p* = 0.001137 → Anodal (c) significantly different from all groups (a, b).

LT Contact Score: H = 24, *p* = 0.00007987 → Negative control and Cathodal (a) not different; both differ from Untreated, Sham, and Anodal (b).

.

**Table 2:** Removal time in different experimental groups: Values are presented as mean ± SD. - Indicates test failure; cutoff value assigned to rats that failed to complete the test.

Group	Before the Surgery				After the Surgery				RT Sig*	LT Sig*
	RT Removal Time (s)	RT Score	LT Removal Time (s)	LT Score	RT Removal Time (s)	RT Score	LT Removal Time (s)	LT Score		
Negative control	20.14 ± 2.01	1.0 ± 0.0	8.40 ± 0.84	1.0 ± 0.0	24.98 ± 2.50	1.0 ± 0.0	24.98 ± 2.50	1.0 ± 0.0	*	*
Untreated	12.36 ± 1.24	1.0 ± 0.0	13.12 ± 1.31	1.0 ± 0.0	121.00 ± 0.0-	3.0 ± 0.0	121.00 ± 0.0-	3.0 ± 0.0	b	b
Sham	44.18 ± 4.42	2.0 ± 0.0	55.46 ± 5.55	2.0 ± 0.0	121.00 ± 0.0-	3.0 ± 0.0	121.00 ± 0.0-	3.0 ± 0.0	b	b
Anodal	21.40 ± 2.14	1.0 ± 0.0	22.71 ± 2.27	1.0 ± 0.0	121.00 ± 0.0-	3.0 ± 0.0	121.00 ± 0.0-	3.0 ± 0.0	b	b
Cathodal	8.54 ± 0.85	1.0 ± 0.0	9.53 ± 0.95	1.0 ± 0.0	10.96 ± 1.10	1.0 ± 0.0	94.23 ± 9.42	2.5 ± 0.5	*	–

Groups not sharing the same superscript letter differ significantly.

RT Removal Time: H = 24, *p* = 0.00007987 → Negative control differs from all groups except Cathodal; Cathodal differs from all groups except Negative control.

LT Removal Time: H = 24, *p* = 0.00007987 → Negative control differs from all groups except Cathodal.

### TTC staining

**Fig. 4:**
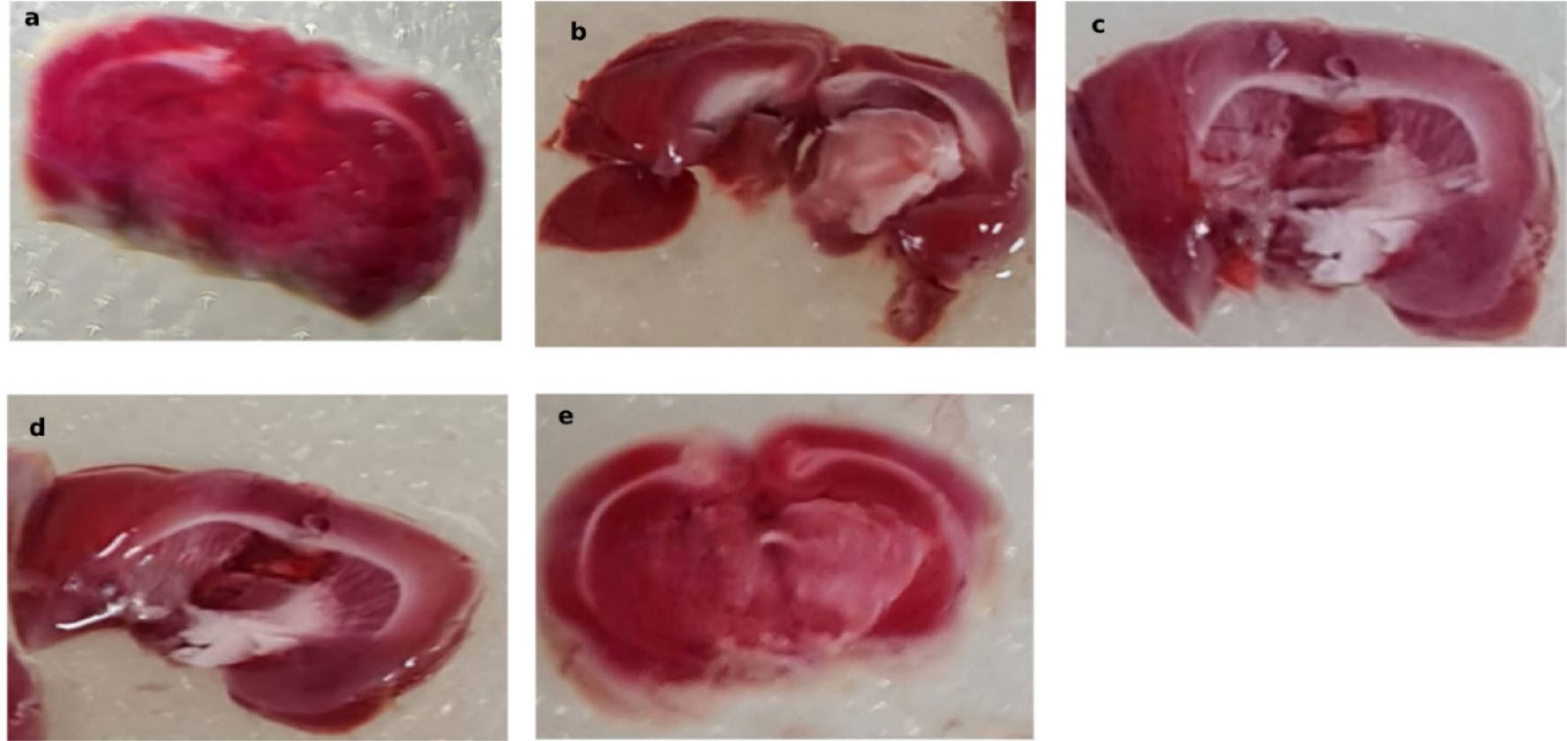
Representative TTC-stained brain sections showing infarcted regions in **(a)** Normal non-ischemic, **(b)** Untreated, **(c)** Sham, **(d)** Anodal, and **(e)** Cathodal groups. Images are presented for descriptive comparison only. TTC analysis was performed on a single representative animal per group, and no quantitative or statistical comparison was conducted.

TTC staining showed infarcted regions as pale unstained areas in ischemic groups, with no visible infarction in the control group (Fig. 4).

### H&E Staining

**Fig. 5:**
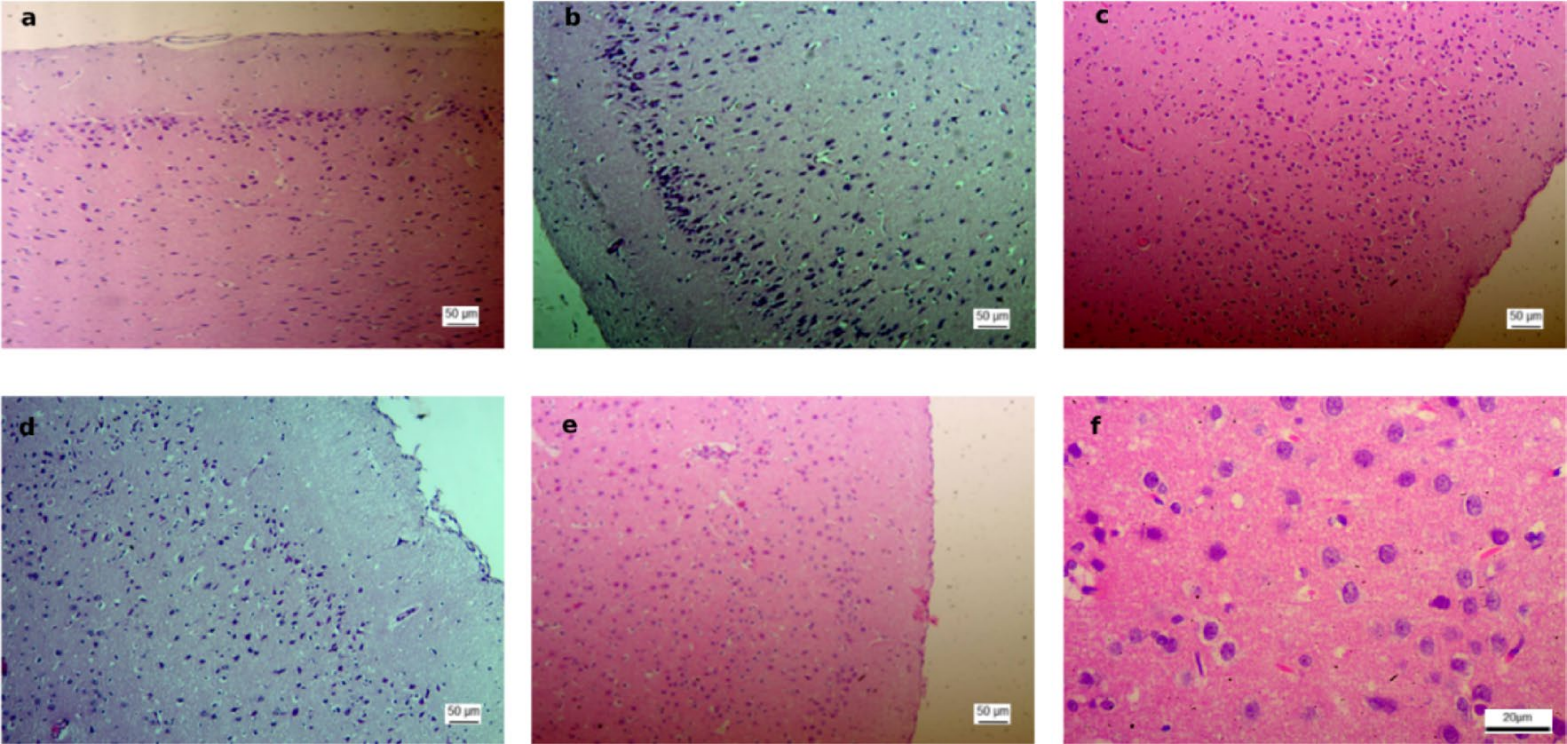
Non-ischemic cortical regions showing preserved neuronal architecture. **(a)** Representative H&E-stained coronal section at ×100 from the Normal non-ischemic group. **(b)** Corresponding ×100 section from the Untreated group. **(c)** ×100 section from the Sham group. **(d)** ×100 section from the Anodal group. **(E)** ×100 section from the Cathodal group. **(F)** Higher magnification (×400) of the Normal non-ischemic group illustrating intact cortical cytoarchitecture with preserved neuronal layers and vesicular nuclei. Scale bars: 50 μm (×100) and 20 μm (×400).

In the Normal non-ischemic group and non-ischemic brain tissues across all stroke groups, histological examination revealed well-preserved architecture, characterized by regular neuronal layers with vesicular nuclei (Fig. 5).

**Fig. 6:**
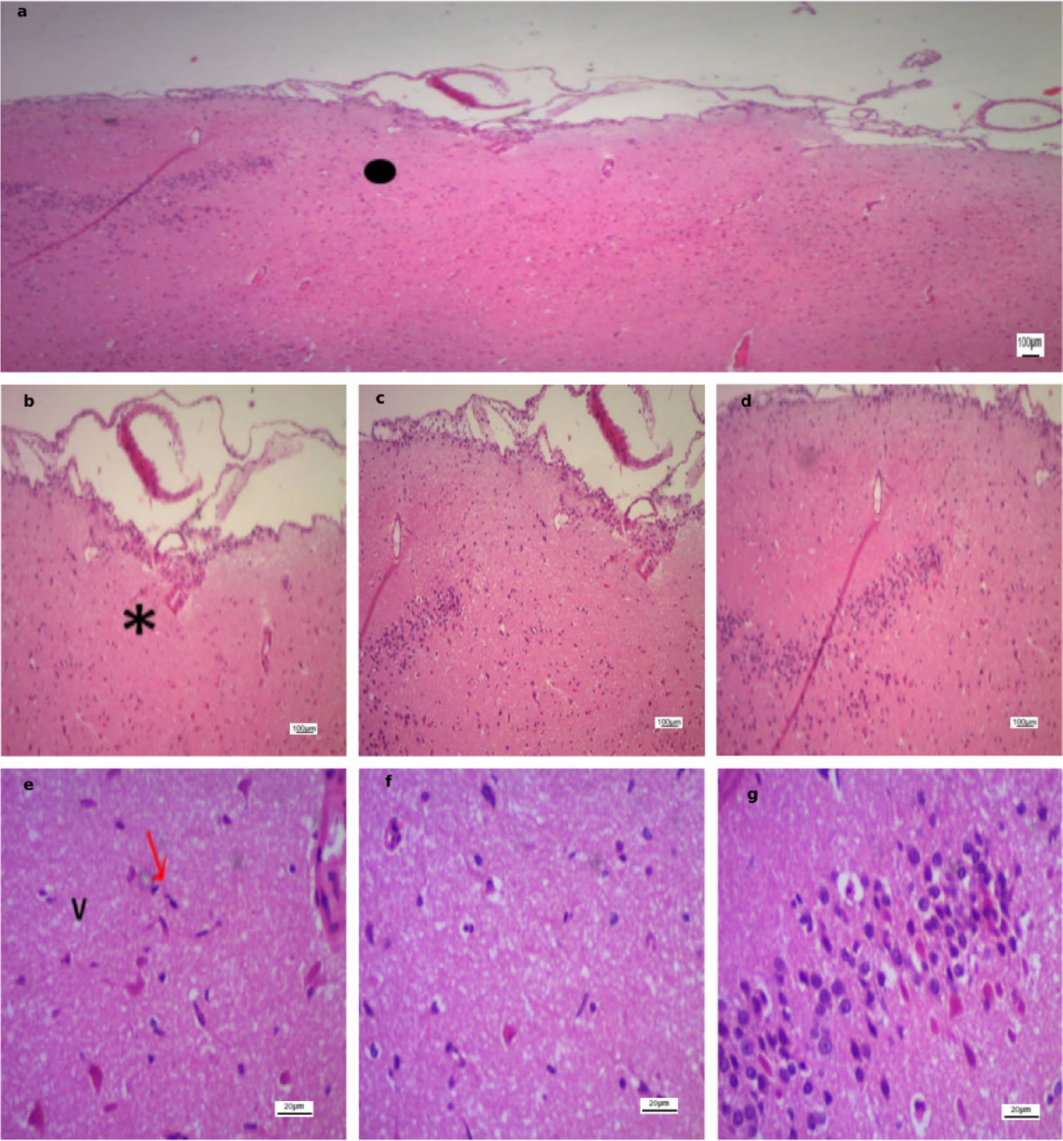
Photomicrographs of ischemic cortical regions in the Untreated group. **(a)** Low-power field (×40) subdivided into Area A (severe ischemic injury with pyknotic nuclei), Area B (moderate ischemic changes), and Area C (preserved cortical architecture). **(b–d)** Higher magnification (×100) from Area A, Area B, and Area C, respectively (same scale bar). **(e–g)** High-power fields (×400) from Area A, Area B, and Area C, respectively, highlighting disrupted cortical layering (), necrosis (*), pyknotic nuclei ($\color{red} {\uparrow}$), and cytoplasmic vacuolation (V). Scale bars: 100 μm **(a–d)**, 20 μm **(e–g)**.

**Fig. 7:**
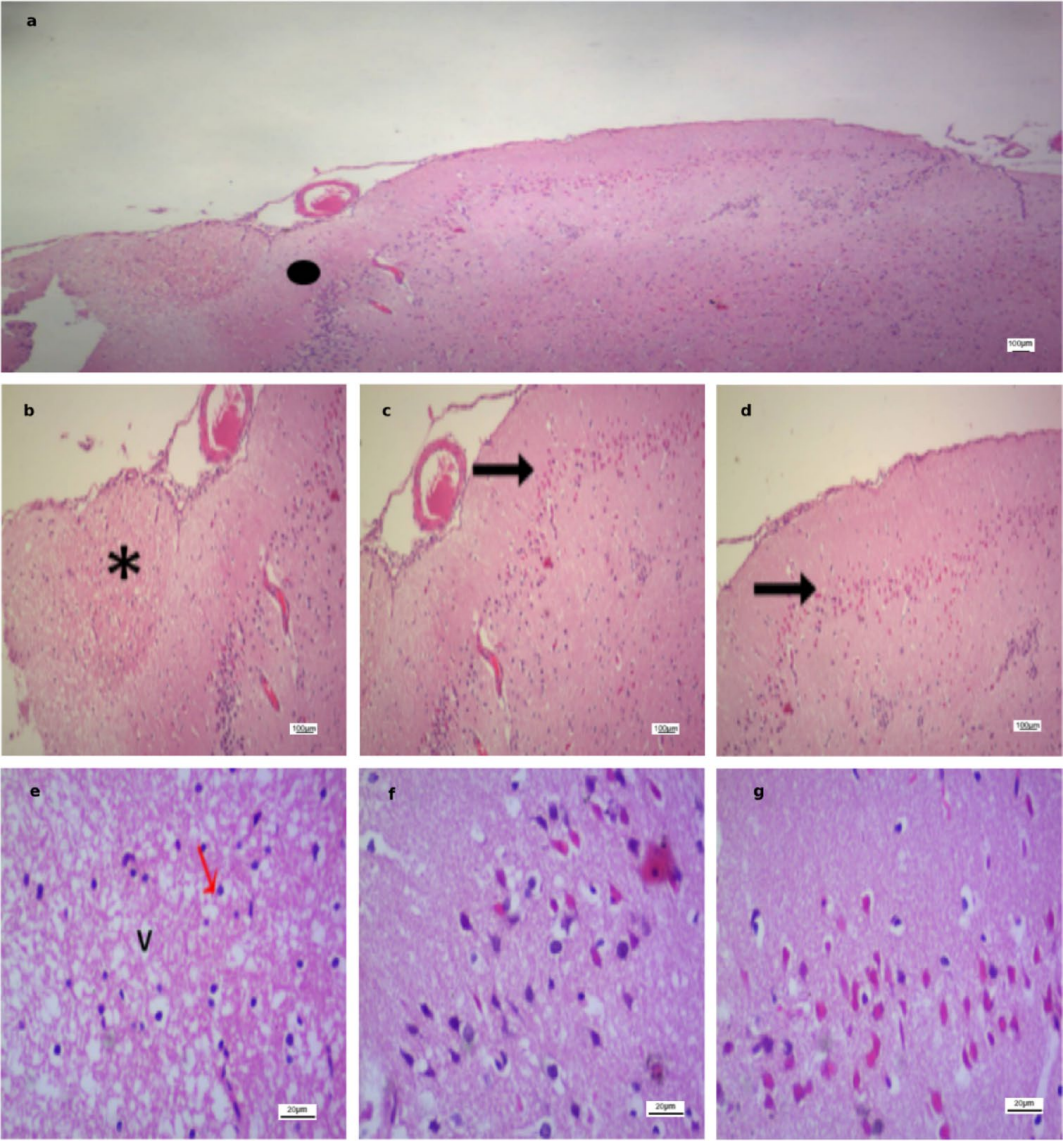
Photomicrographs of ischemic cortical regions in the Sham group. **(a)** Low-power field (×40) subdivided into Area A (severe ischemic injury with pyknotic nuclei), Area B (moderate ischemic changes), and Area C (preserved cortical architecture). **(b–d)** Higher magnification (×100) from Area A, Area B, and Area C, respectively (same scale bar). **(e–g)** High-power fields (×400) from Area A, Area B, and Area C, respectively, highlighting disrupted cortical layering (), necrosis (*), eosinophilic neurons (↑), pyknotic nuclei ($\color{red} {\uparrow}$), and cytoplasmic vacuolation (V). Scale bars: 100 μm **(a–d)**, 20 μm **(e–g)**.

**Fig. 8:**
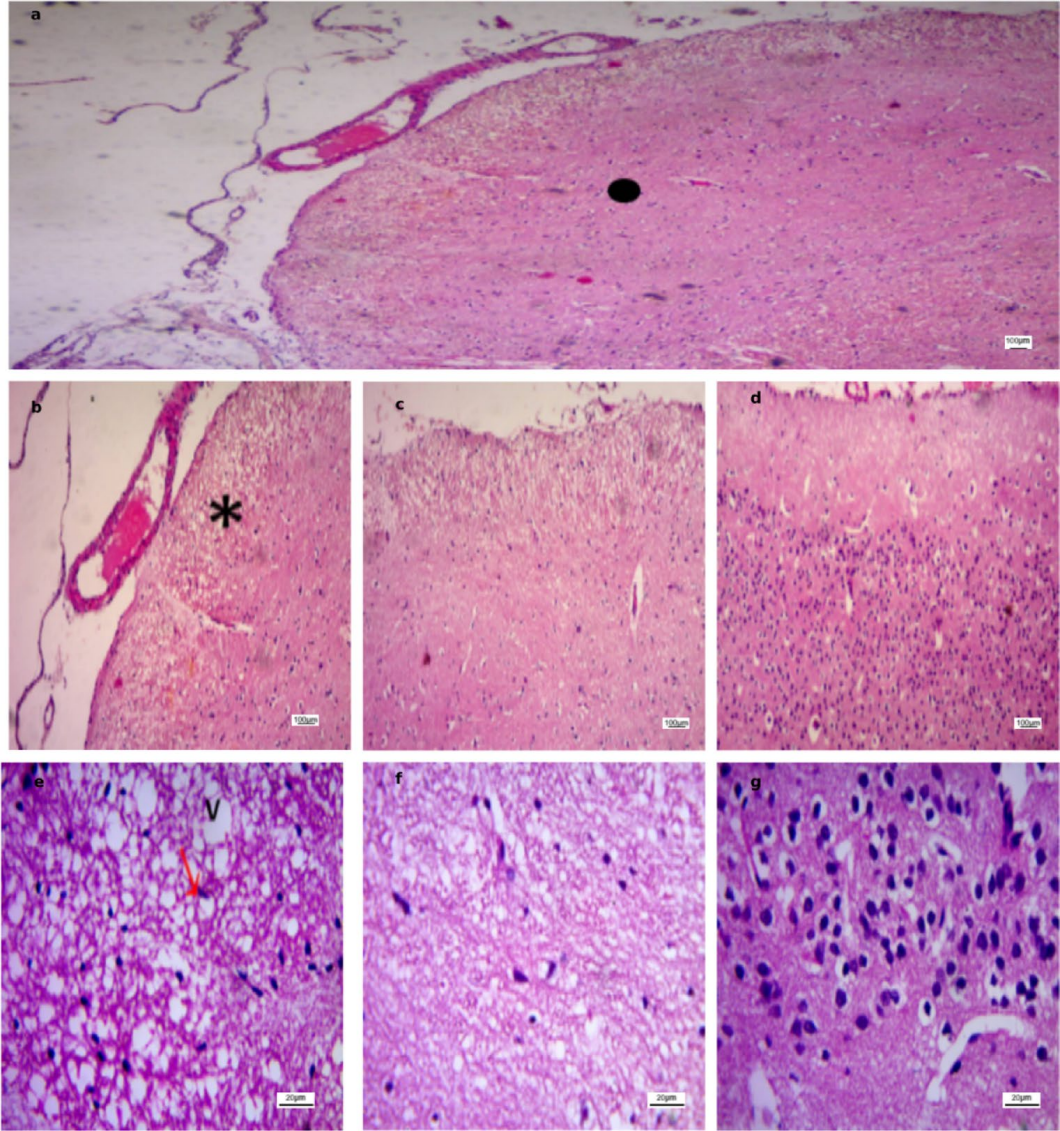
Photomicrographs of ischemic cortical regions in the Anodal group. **(a)** Low-power field (×40) subdivided into Area A (severe ischemic injury with pyknotic nuclei), Area B (moderate ischemic changes), and Area C (preserved cortical architecture). **(b–d)** Higher magnification (×100) from Area A, Area B, and Area C, respectively (same scale bar). **(e–g)** High-power fields (×400) from Area A, Area B, and Area C, respectively, highlighting disrupted cortical layering (), necrosis (*), pyknotic nuclei ($\color{red} {\uparrow}$), and cytoplasmic vacuolation (V). Scale bars: 100 μm **(a–d)**, 20 μm **(e–g)**.

**Fig. 9:**
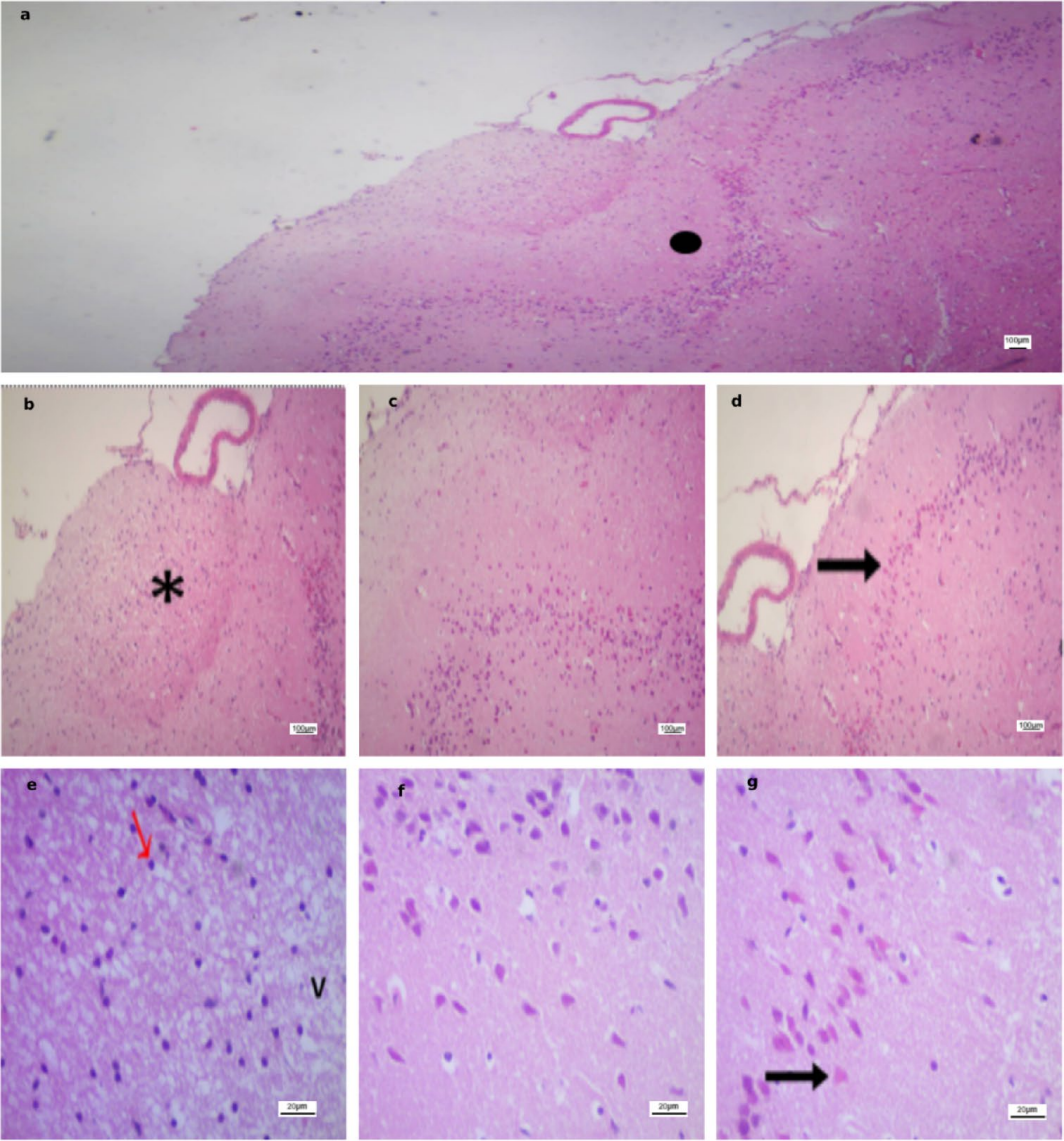
Photomicrographs of ischemic cortical regions in the Cathodal group. **(a)** Low-power field (×40) subdivided into Area A (severe ischemic injury with pyknotic nuclei), Area B (moderate ischemic changes), and Area C (preserved cortical architecture). **(b–d)** Higher magnification (×100) from Area A, Area B, and Area C, respectively (same scale bar). **(e–g)** High-power fields (×400) from Area A, Area B, and Area C, respectively, highlighting disrupted cortical layering (), necrosis (*), eosinophilic neurons (↑), pyknotic nuclei ($\color{red} {\uparrow}$), and cytoplasmic vacuolation (V). Scale bars: 100 μm **(a–d)**, 20 μm **(e–g)**.

In contrast, the stroke-induced groups (Figs. 6, 7, 8 and 9) exhibited marked histopathological alterations. These changes were characterized by disruption of normal cortical lamination and altered hematoxylin and eosin (H&E) staining patterns, consistent with necrosis and extensive neuronal injury. Across all groups, the most frequently observed pathological features included eosinophilic neurons, reflecting early infarction and mitochondrial dysfunction, as well as pyknotic nuclei suggestive of neuronal death, cytoplasmic vacuolation indicative of neuronal edema, and perivascular edema.

Region-specific differences in ischemic severity were also evident. Area A demonstrated the most pronounced injury, characterized by densely pyknotic nuclei and extensive structural disorganization. Area B displayed moderate ischemic alterations, representing an intermediate zone between the severely affected Area A and the relatively preserved Area C. In contrast, Area C maintained intact cortical architecture with well-organized neuronal layers and vesicular nuclei.

**Fig. 10:**
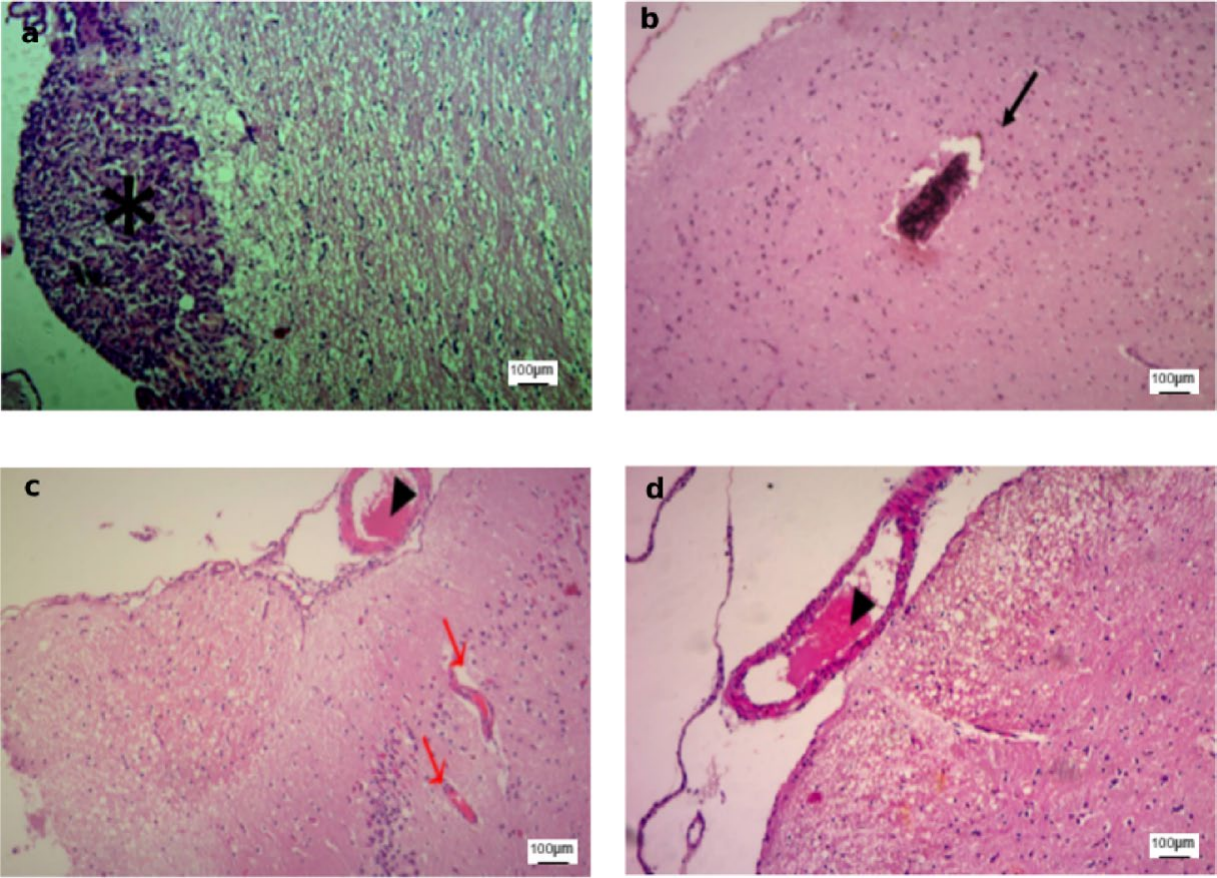
Ischemic cortical areas (H&E, ×100) from untreated, sham, and anodal groups. **(a)** Untreated group with dense rim of cellular infiltration (*). **(b)** Untreated group showing intravascular thrombus (↑). **(c)** Sham group with dilated/congested vessels (▲) and extensive hemorrhage (↑). **(d)** Anodal group with dilated/congested vessels (▲). Scale bar: 100 μm.

Figure 10 demonstrates advanced histopathological alterations in all stroke groups, with the exception of the cathodal tDCS-treated group. In the untreated group, a dense rim of cellular infiltration was evident at the infarct margins, consistent with gliosis and an active inflammatory response, reflecting ongoing tissue remodeling and organization of the injury. Additionally, a darkly stained intraluminal mass composed of compacted erythrocytes and fibrin was identified, nearly occluding the vascular lumen. This lesion most likely represented an intravascular thrombus and was frequently observed within capillaries, indicating a poor prognosis and impaired stroke resolution. In the sham and anodal tDCS groups, blood vessels appeared dilated and congested, and the sham group displayed extensive hemorrhagic regions. Collectively, these findings indicate that cathodal tDCS may attenuate ischemic injury, reduce acute necrosis, and facilitate tissue repair, potentially through modulation of inflammatory and glial responses.

### Nissl staining

**Fig. 11:**
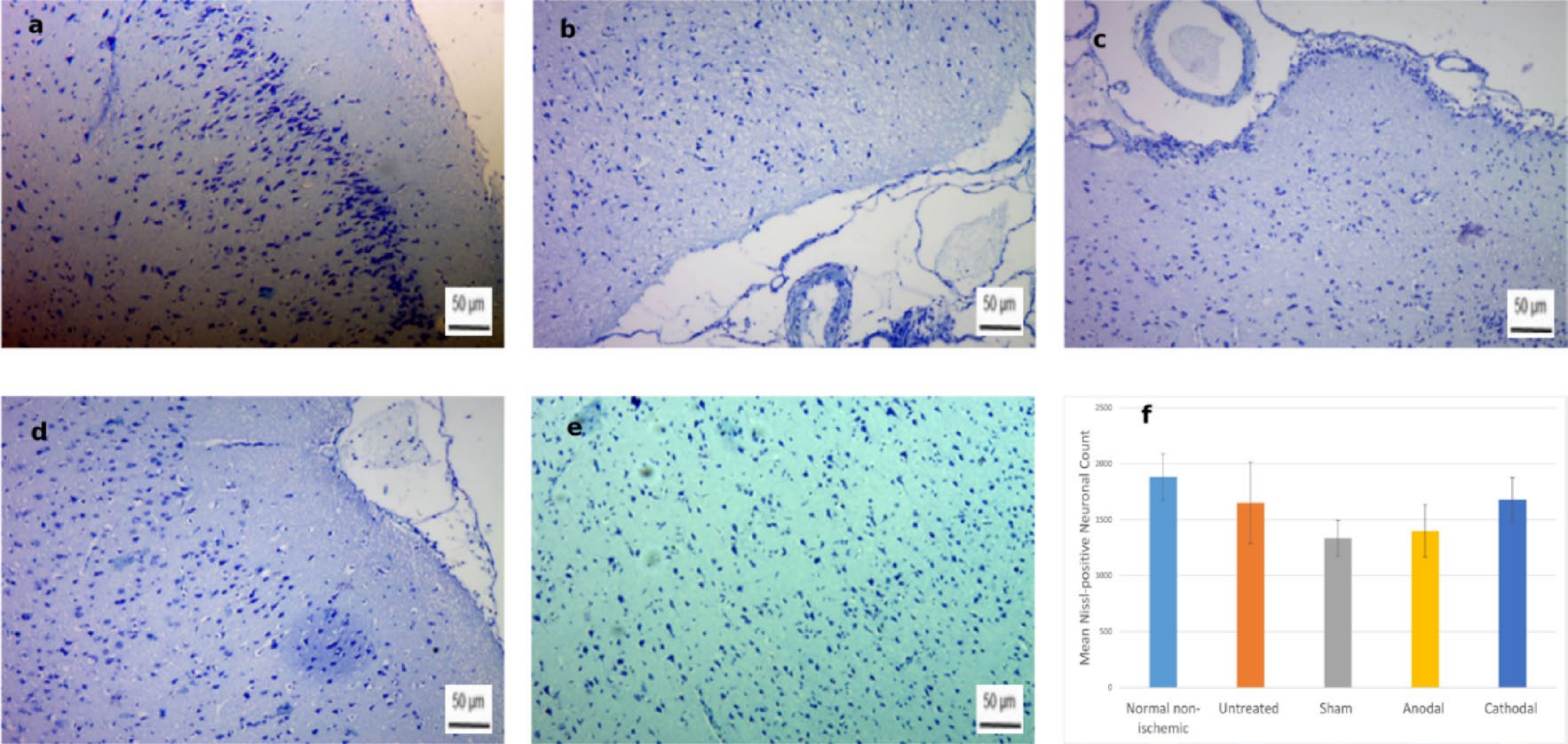
Representative Nissl-stained (Toluidine Blue) brain sections from **(a)** Normal non-ischemic, **(b)** Untreated, **(c)** Sham, **(d)** Anodal, and **(e)** Cathodal groups. **(f)** Quantification of neuronal density shows no significant differences across groups. Scale bar: 50 μm.

Quantitative assessment of Nissl staining revealed no significant group differences (*p* = 0.52,** F = 0.84;** Fig. 11). The Normal non-ischemic group showed the highest mean neuronal count (**1880.75 ± 414.36**), followed by the Cathodal (**1681.75 ± 391.85**) and Untreated (**1649.75 ± 727.99**) groups. The Anodal (**1400.75 ± 470.38**) and Sham (**1334.50 ± 320.92**) groups demonstrated lower mean values. Nissl staining did not show statistically significant differences in neuronal density among the experimental groups, although a trend toward improved neuronal preservation was observed in the cathodal tDCS group.

### TNF-α expression

**Fig. 12:**
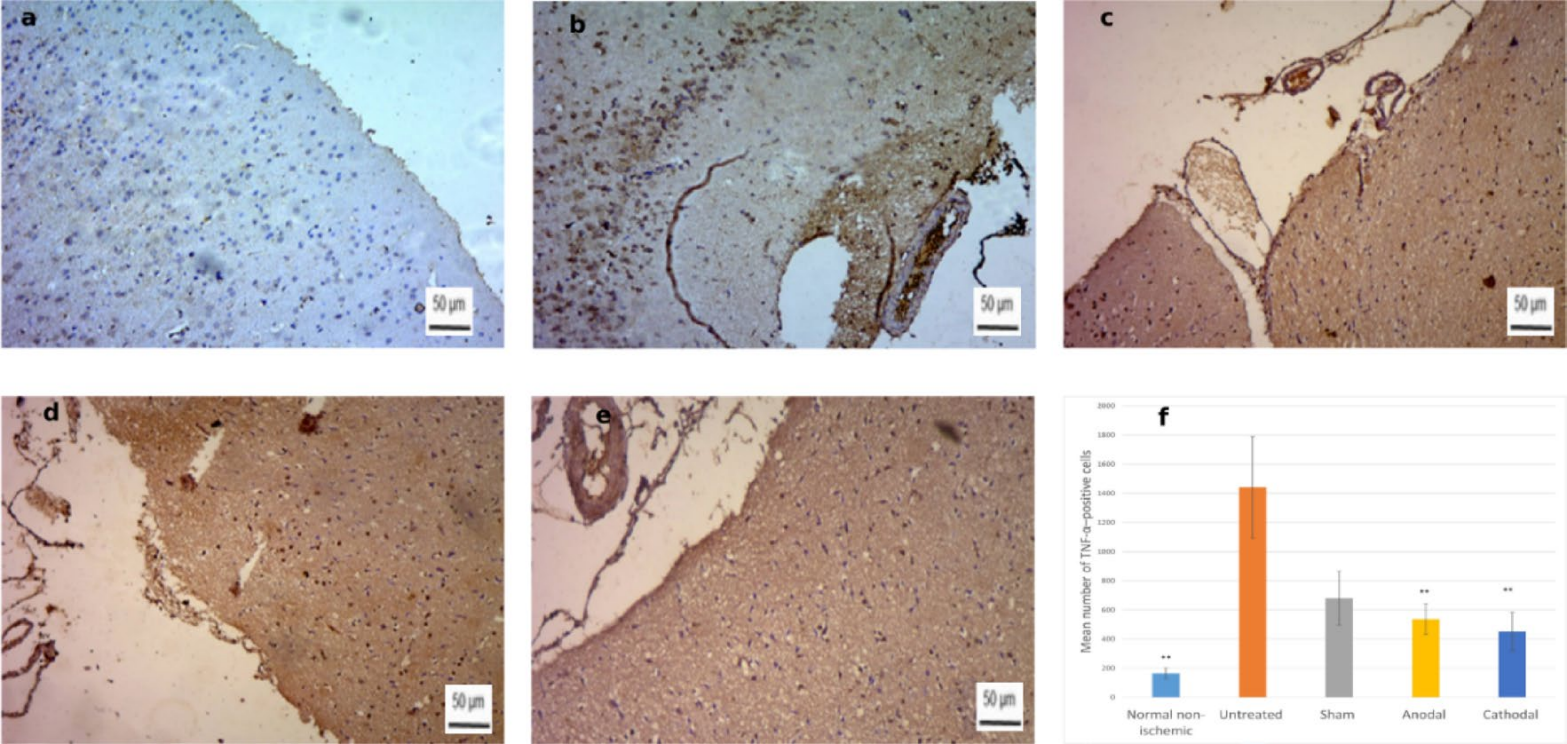
Representative TNF-α–stained brain sections from **(a)** Normal non-ischemic, **(b)** Untreated, **(c)** Sham, **(d)** Anodal, and **(e)** Cathodal groups. **(f)** Quantification shows highest expression in the Untreated group, with Sham and Anodal at intermediate levels, and a significant reduction in the Cathodal group. Scale bar: 50 μm. (**p = 0.0038; Untreated vs. all except Sham).

Quantitative assessment of TNF-α immunoreactivity revealed significant group differences (F = 6.17, *p* = 0.0038; Fig. 12). The Untreated group exhibited the highest mean number of TNF-α–positive cells (1440.50 ± 696.78), which was significantly greater than the Cathodal group (450.50 ± 263.18; *p* = 0.0038). The Sham (681.75 ± 369.28) and Anodal (536.25 ± 211.73) groups showed intermediate values, both significantly lower than the Untreated group but not different from the Cathodal or Normal non-ischemic (164.25 ± 73.44). These results indicate a robust post-ischemic inflammatory response in the absence of treatment and suggest a marked anti-inflammatory effect of cathodal tDCS.

### c-Fos expression

**Fig. 13:**
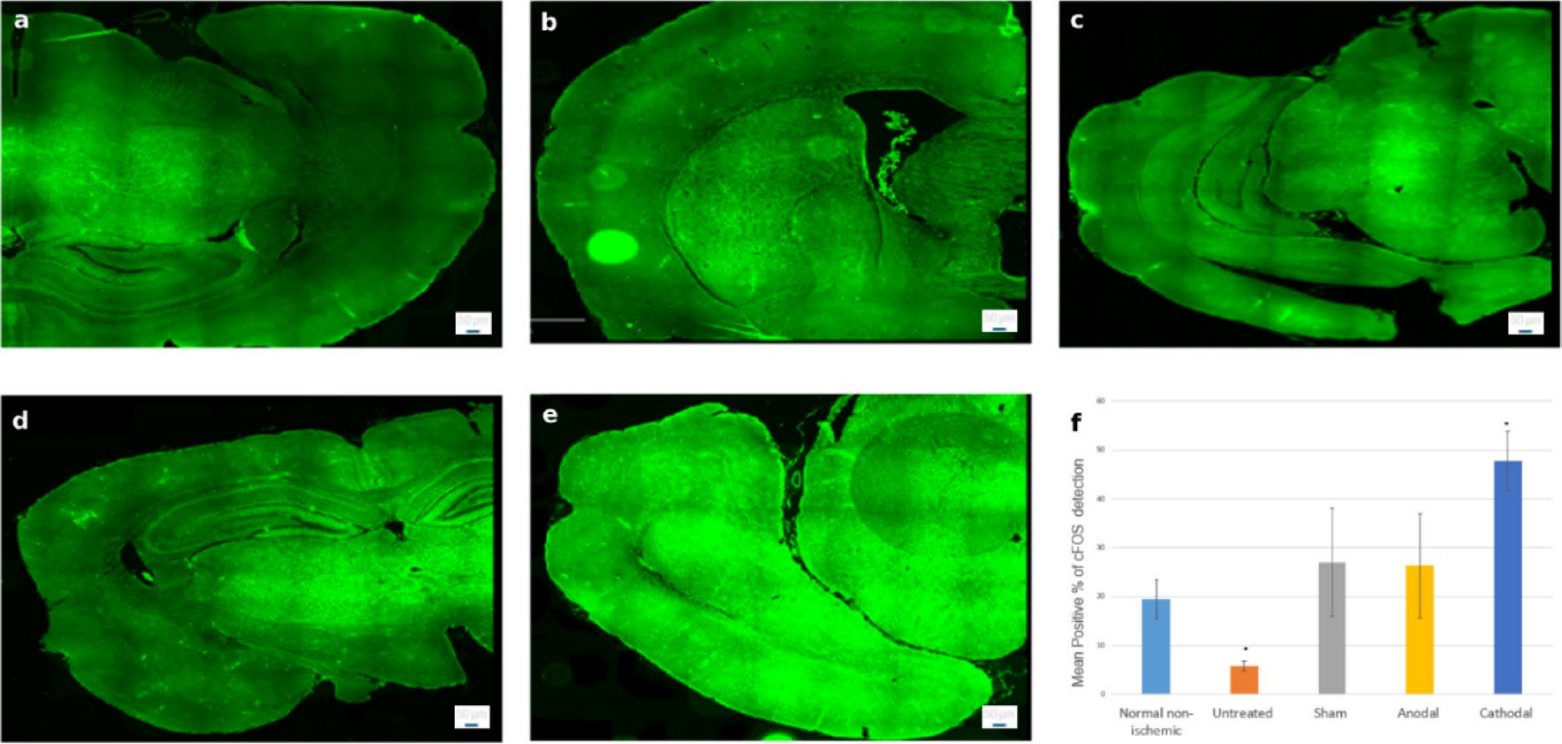
Representative c-Fos–stained brain sections from **(a)** Normal non-ischemic, **(b)** Untreated, **(c)** Sham, **(d)** Anodal, and **(e)** Cathodal groups. **(f)** Quantification shows highest expression in the Cathodal group compared with Untreated, with Sham and Anodal at intermediate levels. Scale bar: 50 μm. *p = 0.0218 (Untreated vs. Cathodal).

Quantitative assessment of c-Fos immunoreactivity revealed significant group differences (*p* = 0.02, F = 3.96; Fig. 13). The Cathodal stimulation group demonstrated the highest proportion of c-Fos-positive cells (47.77 ± 12.31%), which was significantly greater than the Untreated group (5.87 ± 2.07%; *p* = 0.02). The Sham (27.03 ± 22.04%) and Anodal (26.40 ± 21.33%) groups exhibited intermediate values, which did not differ significantly from either Cathodal or Normal non-ischemic (19.48 ± 8.08%). These findings highlight the temporal dynamics of c-Fos expression and the potential of cathodal tDCS to enhance neuronal activation in the acute phase following ischemic stroke.

### CD206 expression

**Fig. 14:**
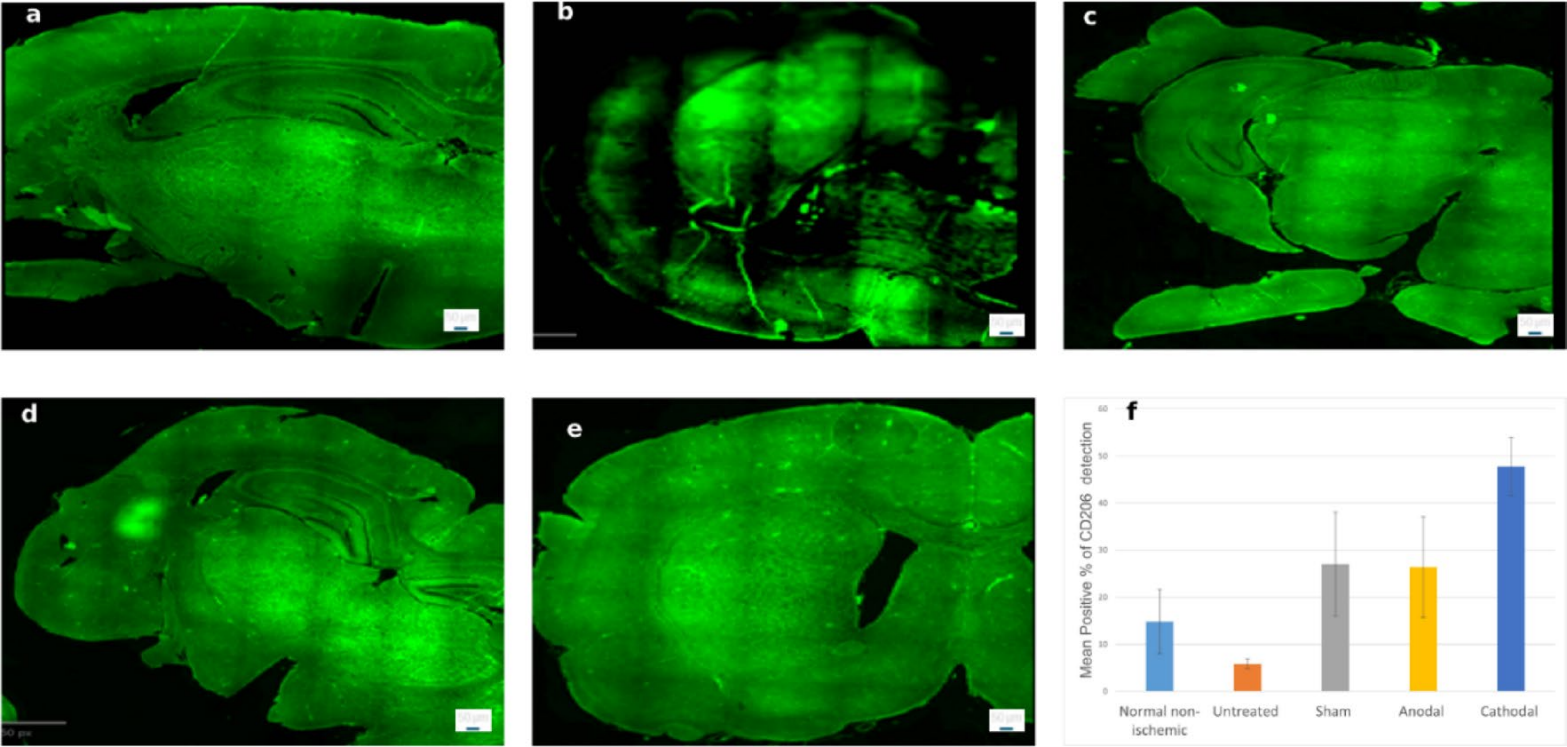
Representative CD206–stained brain sections from **(a)** Normal non-ischemic, **(b)** Untreated, **(c)** Sham, **(d)** Anodal, and **(e)** Cathodal groups. **(f)** Quantification shows the highest expression in the Cathodal group, with Sham and Anodal at intermediate levels, Untreated showing comparable expression, and Normal non-ischemic the lowest. Differences were not statistically significant. Scale bar: 50 μm.

Quantitative assessment of CD206 immunoreactivity revealed no significant group differences (F = 0.7767, *p* = 0.5573; Fig. 14). The Cathodal stimulation group demonstrated the highest proportion of CD206-positive cells (28.57 ± 16.76%), followed by the Anodal (30.32 ± 15.03%) and Sham (27.98 ± 11.47%) groups, which showed comparable intermediate values. The Untreated group (30.28 ± 16.66%) also exhibited similar expression levels, whereas the Normal non-ischemic group demonstrated the lowest proportion (14.82 ± 13.76%). CD206 expression did not demonstrate statistically significant differences between groups. However, a non-significant increase in CD206-positive cells was observed in the cathodal tDCS group.

### GFAP expression

**Fig. 15:**
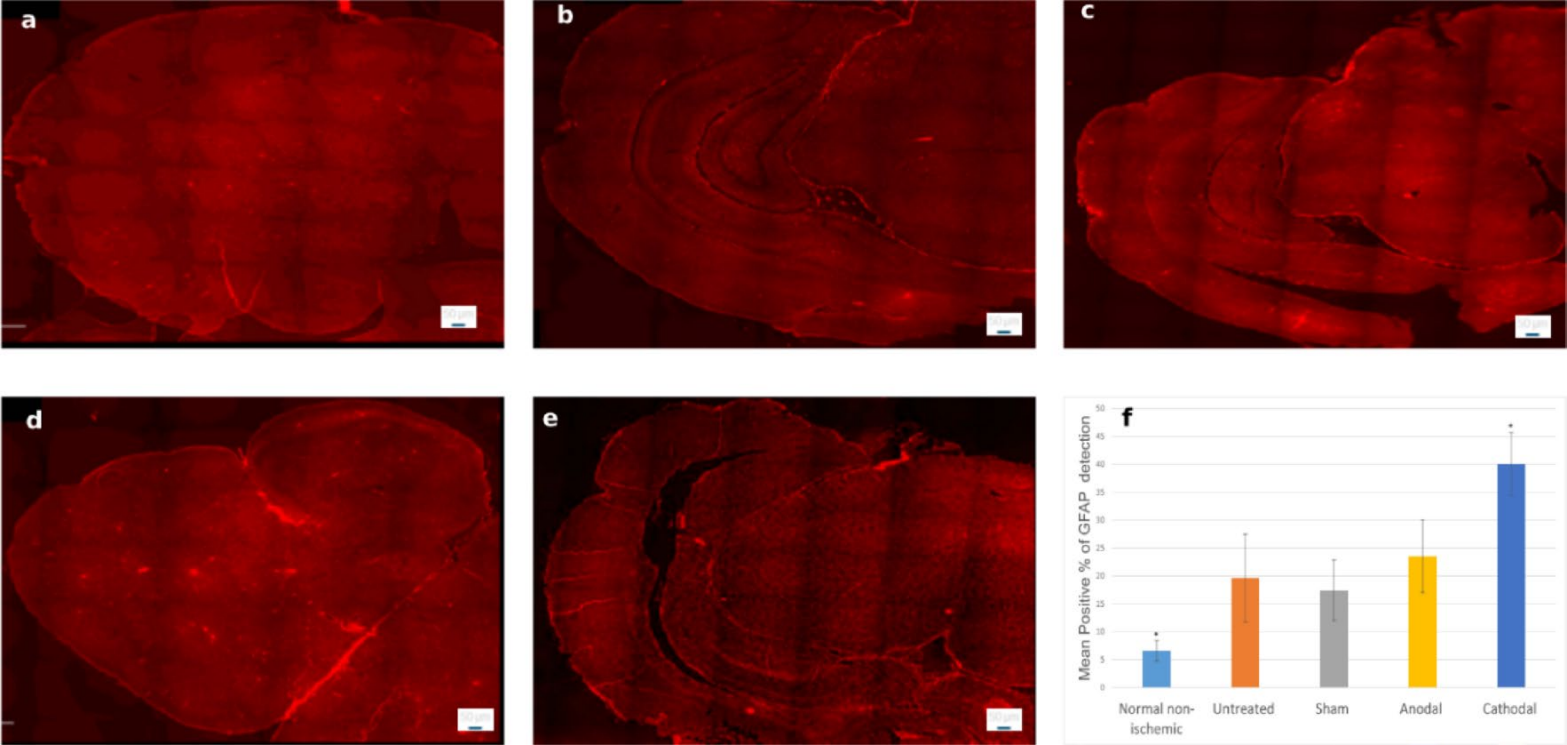
Representative GFAP–stained brain sections from **(a)** Normal non-ischemic, **(b)** Untreated, **(c)** Sham, **(d)** Anodal, and **(e)** Cathodal groups. **(f)** Quantification shows highest expression in the Cathodal group (~ 40%) compared with the Normal non-ischemic (~ 6%; *p* = 0.015), with Sham (~ 18%) and Anodal (~ 23%) at intermediate levels, and Untreated at ~ 20% (F = 4.37). Scale bar: 50 μm. *p = 0.0154 (Cathodal vs. Normal non-ischemic).

Quantitative assessment of GFAP immunoreactivity revealed significant group differences (*p* = 0.015, F = 4.37; Fig. 15). The Cathodal stimulation group demonstrated the highest proportion of GFAP-positive cells (40.03 ± 11.31%), which was significantly greater than the Normal non-ischemic group (6.56 ± 3.70%). The Anodal (23.51 ± 13.00%) and Sham (17.42 ± 10.85%) groups exhibited intermediate values, while the Untreated group (19.61 ± 15.77%) also showed elevated expression relative to the Normal non-ischemic. These findings highlight the progressive increase in GFAP expression following cathodal stimulation and suggest the potential of stroke-induced astrocytic activation in the acute phase of ischemic injury.

### Study losses and animal replacement

A total of eleven rats were lost due to postoperative complications, and replacement rats were added to maintain group balance (initial sample size = 5 per group). Postoperative mortality distribution was: Untreated (3/5; 60%), Anodal (5/5; 100%), Sham (2/5; 40%), and Cathodal (1/5; 20%). The Cathodal group exhibited the lowest postoperative mortality rate.

## Discussion

In this study, we found polarity-specific effects of transcranial direct current stimulation (tDCS) in a rat model of acute ischemic stroke. Cathodal stimulation conferred significant neuroprotection, promoting neurological recovery, and preserving sensorimotor function. At the cellular level, cathodal stimulation attenuated neuroinflammation, and promoted early astrocytic activation. These glial adaptations appear to support tissue repair and likely contribute to the improved neurological outcomes observed.

In contrast, anodal stimulation was consistently detrimental. Behaviorally, anodal-treated rats exhibited impaired recovery, with partial dysfunction on the right side and severe deficits on the left side in the adhesive removal test. Many animals failed to complete the task, and several developed comas in the acute phase, underscoring the absence of functional benefit. Histologically, the anodal group demonstrated dilated, congested vessels and lower neuronal survival compared with cathodal and stroke groups. At the molecular level, anodal stimulation induced only intermediate changes, with elevated TNF-α expression indicating a persistent inflammatory response, and moderate increases in c-Fos and GFAP immunoreactivity that failed to translate into improved outcomes. Taken together, these findings suggest that anodal tDCS not only lacked therapeutic benefit but also exacerbated post-stroke deficits, whereas cathodal tDCS promoted neuroprotection through modulation of glial responses and enhancement of reparative pathways.

The effects of transcranial direct current stimulation in ischemic stroke remain variable and depend on stimulation parameters and timing, with many previous studies focusing on delayed application or a single polarity^14,15^. In contrast, the present study investigates the hyperacute phase and directly compares cathodal and anodal stimulation under identical conditions, demonstrating the potential of early, short-duration protocols. These findings contribute to optimizing stimulation strategies and enhancing their translational relevance in acute stroke.

The interpretation of c-Fos expression in the context of tDCS requires careful consideration. Although c-Fos is widely regarded as a marker of neuronal activation, its expression does not necessarily indicate increased excitotoxicity, particularly in complex pathological conditions such as acute ischemic stroke^25^.

Recent evidence suggests that the effects of transcranial direct current stimulation are highly dependent on neuronal orientation, morphology, and local circuit dynamics. For instance, studies have demonstrated that tDCS can induce heterogeneous, polarity-dependent modulation of neuronal firing, where the direction of activity change varies according to the somatodendritic orientation of neurons relative to the applied electric field^15^.

Therefore, the observed increase in c-Fos expression following cathodal stimulation in the present study may reflect selective or network-level modulation of neuronal activity, rather than a uniform increase in excitability or excitotoxic damage. This highlights the complexity of tDCS-induced neuromodulation and underscores the need for cautious interpretation of activity-dependent markers in this context.

The behavioral improvement observed in the cathodal group aligns with this nuanced view of c-Fos expression, suggesting that enhanced functional outcomes may be underpinned by early modulation of neuronal activity, which can prevent hyperexcitability and excitotoxic injury. As an immediate early gene and marker of synaptic plasticity, c-Fos reflects activity-dependent transcriptional responses linked to network reorganization^26^.

This is consistent with models of homeostatic plasticity, where early inhibitory modulation primes neurons for subsequent adaptive remodeling via calcium-dependent transcription factors and MAPK/ERK signaling cascades^27^. The paradoxical observation that cathodal tDCS—typically inhibitory in healthy cortex—promoted recovery after ischemia suggests that pathological tissue alters the polarity-dependent responses to stimulation. Ischemic neurons, experiencing disrupted ionic gradients, membrane depolarization, and excitotoxic stress, may benefit from cathodal currents that stabilize hyperexcitable membranes, reduce calcium influx, and protect penumbral neurons^28^. Conversely, anodal stimulation may exacerbate excitotoxicity by further increasing neuronal excitability, consistent with the poorer outcomes observed in the anodal group.

Moreover, mitochondria have been identified as critical targets of non-invasive stimulation in the ischemic brain, given their central role in oxidative stress and energy failure. In this context, cathodal tDCS may promote neuroprotection by preserving mitochondrial integrity, reducing ROS generation, and sustaining cellular bioenergetics, thereby reinforcing its anti-excitotoxic and anti-inflammatory actions^29^.

Our histological and molecular findings provide mechanistic support for this interpretation. Cathodal tDCS significantly reduced TNF-α expression, indicating suppression of pro-inflammatory cascades that drive secondary neuronal death^30^. Attenuation of necrosis and inflammatory infiltration, along with non-significant preservation of Nissl granules, further confirm its protective cellular effects.

Increased GFAP expression suggests astrocytic activation, which may serve as an adaptive gliosis stabilizing the extracellular environment and limiting lesion expansion. Activated astrocytes reduce excitotoxicity by clearing excess glutamate, release neurotrophic factors (e.g., GDNF, CNTF), and support neurovascular integrity. Cathodal tDCS may enhance these protective functions via the JAK/STAT3 pathway, promoting cell survival, anti-inflammatory signaling, and extracellular matrix remodeling, thereby facilitating tissue repair and restricting damage^31^.

Although trends toward increased neuronal preservation and M2 microglial marker expression were observed in the cathodal tDCS group, these differences did not reach statistical significance. Therefore, these findings should be interpreted with caution and may suggest potential effects that require further investigation with larger sample sizes.

M2 microglia secrete anti-inflammatory cytokines and trophic factors, facilitating tissue repair and functional recovery^32^. The combination of reduced pro-inflammatory signaling, beneficial glial responses, and modulation of neuronal activation likely underlies the observed functional benefits of cathodal stimulation.

Recent evidence indicates that tDCS can influence post-ischemic neuroinflammation, reduce microglial reactivity, and enhance neuroplastic responses. In general, anodal currents are excitatory, whereas cathodal stimulation may suppress pathological hyperexcitability and oxidative stress in peri-infarct regions. Glial responses after stroke are also known to vary by brain region, particularly within the penumbra, where astrocytic and microglial activation contribute to secondary injury processes^33^. Although the current study did not separate core and penumbral zones, the cathodal group showed reduced TNF-α expression, non-significant increased CD206 reactivity, and elevated c-Fos levels, consistent with attenuation of inflammatory cascades and promotion of reparative and plasticity-related pathways within vulnerable cortical areas.

Hematoxylin and Eosin staining highlighted the cellular and vascular impact of ischemia and the neuroprotective effects of cathodal tDCS. Stroke groups showed eosinophilic neurons, pyknotic nuclei, cytoplasmic vacuolation, perivascular edema, and dense inflammatory infiltration, reflecting neuronal necrosis, BBB disruption, astrocytic swelling, and gliosis. In contrast, the cathodal group demonstrated attenuation of ischemic damage, with reduced edema, less dense inflammatory infiltration, and comparatively preserved vascular architecture, although some pathological changes were still evident. These findings suggest that cathodal tDCS may support neuronal viability, stabilize the blood–brain barrier, modulate inflammatory responses, and maintain vascular integrity, potentially through nitric oxide signaling and mitigation of oxidative damage^34^.

Cathodal tDCS showed a robust safety profile, reflected by lower mortality and preserved acute-phase neurological function. Its neuroprotection likely involves multiple mechanisms: stabilization of astrocytic aquaporin-4 and the blood–brain barrier reducing edema, mitigation of oxidative stress and endothelial injury limiting hemorrhagic risk, and nitric oxide–mediated vasodilation enhancing microvascular perfusion^35^. Behaviorally, cathodal-treated rats maintained feeding and systemic stability, unlike other groups with severe neurological decline.

While the present study applies stimulation at an ultra-early time point (20 min post-stroke), this should primarily be interpreted as an experimental approach to investigate early pathophysiological mechanisms rather than a directly applicable clinical protocol. In clinical settings, this time window may not be feasible due to the priority of acute diagnostic and reperfusion interventions.

Future clinical studies may explore the optimization of early post-stroke intervention timing for non-invasive brain stimulation in carefully controlled clinical settings.

The use of male animals exclusively represents a limitation of the present study. Contemporary evidence demonstrates that female animals do not exhibit greater variability than males, and that the longstanding assumption of increased variability in females is not supported by experimental data^36,37^.

In addition, sex-specific differences in stroke pathophysiology and treatment response are well established and clinically relevant. Furthermore, the concept of the “variable female” has been critically re-evaluated, with emphasis on potential biases introduced by male-only research designs^38^.

Therefore, the exclusion of female animals limits the generalizability and translational relevance of the findings, particularly given the clinical burden of stroke in women. Future studies should include both sexes to improve reproducibility and applicability.

Another limitation of the present study is the short observation period, as outcomes were assessed only at 24 h post-ischemia. While this time point is relevant for evaluating acute neuronal injury and early inflammatory responses, it does not provide information regarding long-term functional recovery, neuroplasticity, or sustained therapeutic effects.

Future studies incorporating longer follow-up periods are needed to better understand the long-term impact of transcranial direct current stimulation on stroke outcomes.

TTC staining was performed on a single representative animal per group and is presented for descriptive visualization of infarct localization. Due to the limited sample size, no statistical analysis or quantitative comparison was carried out. Therefore, these findings should be interpreted with caution and are considered preliminary, and they were not used to support the main conclusions of the study.

Another limitation of this study is the relatively small sample size (*n* = 5 per group), which may reduce statistical power, particularly in the context of the inherent variability of experimental stroke models. Although this sample size is comparable to that used in similar preclinical studies^3^, larger cohorts would provide more robust and generalizable results. Future studies with increased sample sizes are warranted to confirm and extend the present findings.

Although some representative images may not fully convey the differences between experimental groups, the quantitative analyses performed on multiple sections per animal using automated and standardized protocols provide robust and reproducible evidence supporting the reported findings.

From a translational perspective, these results are important. Ischemic stroke remains a leading cause of death and disability worldwide^1^, with treatment disparities especially pronounced in low- and middle-income countries due to limited access to reperfusion therapies^2^. Non-invasive and low-cost approaches such as tDCS could represent valuable adjunctive therapies. Notably, the recent TRANSPORT2 trial demonstrated that although tDCS combined with rehabilitation did not significantly outperform sham stimulation, the intervention was safe, well-tolerated, and feasible; importantly, the investigators highlighted that delays in diagnosis and patient transfer may have limited efficacy^39^. These observations underscore the need for further studies to optimize stimulation protocols, confirm efficacy across different populations, and establish tDCS as a practical adjunctive therapy in resource-limited healthcare systems.

It is important to acknowledge that post-stroke microglial reactivity is highly diverse and cannot be fully captured by the traditional M1/M2 classification. Recent research shows that microglia can shift into several distinct reactive states, each with unique molecular and functional signatures. A more detailed characterization using microglia-specific markers such as TMEM119 or P2RY12, or advanced approaches like single-cell analysis, would provide a clearer understanding of which immune populations are modified by tDCS^40^.

In conclusion, cathodal tDCS promotes neuroprotection in acute ischemic stroke by attenuating neuroinflammation, stabilizing excitability, and enhancing beneficial astrocytic and suggestive microglial responses. These findings support its translational potential as an adjunctive therapy in the acute phase of stroke, though future work is required to refine stimulation parameters and validate efficacy in clinical settings.

## Data Availability

Availability of data and materialsThe datasets generated and/or analyzed during the current study are not publicly available due to institutional policy and data privacy restrictions but are available from the corresponding author on reasonable request.
